# Palladium Nanoparticles on Chitosan-Coated Superparamagnetic Manganese Ferrite: A Biocompatible Heterogeneous Catalyst for Nitroarene Reduction and Allyl Carbamate Deprotection

**DOI:** 10.3390/polym15010232

**Published:** 2023-01-01

**Authors:** Mona Ebadi, Nurul Asikin-Mijan, Mohd Suzeren Md. Jamil, Anwar Iqbal, Emad Yousif, Ahmad Rifqi Md Zain, Tengku Hasnan Tengku Aziz, Muhammad Rahimi Yusop

**Affiliations:** 1 Department of Chemical Sciences, Faculty of Science and Technology, Universiti Kebangsaan Malaysia (UKM), Bangi 43600, Selangor, Malaysia; monaebadi@ukm.edu.my (M.E.); nurul.asikin@ukm.edu.my (N.A.-M.); suzeren@ukm.edu.my (M.S.M.J.); 2 School of Chemical Sciences, Universiti Sains Malaysia, Penang 11800, Malaysia; anwariqbal@usm.my; 3 Department of Chemistry, College of Science, Al-Nahrain University, Baghdad 64074, Iraq; emad_yousif@hotmail.com; 4 Institute of Microengineering and Nanoelectronics (IMEN), Universiti Kebangsaan Malaysia (UKM), Bangi 43600, Selangor, Malaysia; hasnanaziz@ukm.edu.my

**Keywords:** chitosan, magnetite, palladium magnetite, nitroarene reduction, allyl carbamate deprotection

## Abstract

Although metallic nanocatalysts such as palladium nanoparticles (Pd NPs) are known to possess higher catalytic activity due to their large surface-to-volume ratio, however, in nanosize greatly reducing their activity due to aggregation. To overcome this challenge, superparamagnetic chitosan-coated manganese ferrite was successfully prepared and used as a support for the immobilization of palladium nanoparticles to overcome the above-mentioned challenge. The Pd-Chit@MnFe_2_O_4_ catalyst exhibited high catalytic activity in 4-nitrophenol and 4-nitroaniline reductions, with respective turnover frequencies of 357.1 min^−1^ and 571.4 min^−1^, respectively. The catalyst can also be recovered easily by magnetic separation after each reaction. Additionally, the Pd-Chit@MnFe_2_O_4_ catalyst performed well in the reductive deprotection of allyl carbamate. Coating the catalyst with chitosan reduced the Pd leaching and its cytotoxicity. Therefore, the catalytic activity of Pd-Chit@MnFe_2_O_4_ was proven to be unrestricted in biology conditions.

## 1. Introduction

In the field of catalysis, metallic nanocatalysts are known to possess higher catalytic activity due to their large surface-to-volume ratio [1,2,3]. To illustrate, gold nanoparticles NH_2_-MIL-101(Fe) with easier biodegradability, superior catalytic activity, and less toxicity present promising strategies for industrial wastewater treatment [4]. Other researchers, R.T. Ledari et al., provided silver nanoparticles as a catalyst. These nanoparticles stabilized with volcanic pumice and chitosan and were acknowledged as higher-yield biomolecules in industrial applications [5]. However, nanosized-catalysts are usually involved in complicated isolation and recovery processes, as well as activity loss due to their aggregation [6,7,8]. Therefore, the constraints posed by nanosized-catalysts have become a challenge in the application of transition metals as a catalyst including palladium nanoparticles (Pd NPs). Palladium as a primary catalyst has been widely used in numerous applications. Several studies have been carried out to overcome the challenges involved in the use of Pd NPs as a catalyst by immobilizing the nanosized catalyst on insoluble metal supports such as silica [9,10], carbon [11,12], and polymeric materials [13,14,15]. For instance, I. Sargin et al. reported that the preparation of chitosan-carbon nanotube-supported palladium catalyst nanoparticles are efficient in reducing nitroarenes in industrial effluents [16]. Further studies demonstrated that the catalytic potential of the Pd-graphene oxide nanocomposite was effective for the degradation of environmental contaminants [17]. Additionally, N-maleyl chitosan-supported palladium as an environmentally friendly catalyst exhibited high catalytic and economic performance [18].

In previous studies, magnetic materials were considered ideal supports for heterogeneous catalysts [19,20]. J. Rahimi et al. reported an organic–inorganic hybrid nanocomposite constructed of polyvinyl alcohol, iron oxide, and silver nanoparticles, which is a promising selection for an efficient catalytic system. They found that this nanocomposite has great catalytic performance, with high yields in short times for biological activity [21]. The magnetic-supported catalysts can be conveniently and easily recovered through the magnetic separation technique using an external magnetic field [22]. R.T. Ledari et al. reported a magnetic catalyst constructed of iron oxide and silver nanoparticles with an isothiazolone organic structure used for the conversion of nitrobenzene derivatives to their aniline forms. These nanoparticles are efficient catalysts with high magnetic properties and excellent reusability [23]. Moreover, F. H. Afruzi introduced a novel mesoporous nanocomposite that was magnetized by magnetite nanoparticles (APTES@SBA-15/Fe_3_O_4_), which exhibited excellent catalytic activity [24]. A wide range of magnetic catalysts are used for industrial purposes. For example, Sh. Bahrami et al. designed the magnetic biocompatible rod-like ZnS/CuFe_2_O_4_/agar organometallic hybrid catalyst with notable strengths [25]. Among the magnetic materials, nanoparticles of manganese ferrite (MnFe_2_O_4_), which is a type of spinel ferrite (MFe_2_O_4_, where M(II) is a d-block transition metal), have attracted much interest due to their high saturation magnetization (M_s_) [26]. Moreover, MnFe_2_O_4_ usually exhibits superparamagnetic behaviour below the dimensions of 50 nm [27]. The superparamagnetic properties of MnFe_2_O_4_ allow it to be easily separated using a magnet and can be redispersed in the absence of an external magnetic field [28].

Coating magnetic nanoparticles with non-toxic materials prevents the degradation of their magnetic properties during catalytic reactions [29] and reduces the toxicity of toxic ferrite nanoparticles [30]. Among the various coating materials, biopolymers have gained much attention due to environmental issues [31]. Chitosan is one example of an eco-friendly biopolymer which exists abundantly in nature and can be obtained from organic waste at low production cost [32]. Besides that, chitosan is an ideal biopolymer for heterogeneous catalyst preparation because it only dissolves in acidic conditions but is insoluble in water and organic solvent [33]. Previous studies in the literature have reported that the amine functional group of chitosan can promote metal ion chelation [34]. This unique feature makes chitosan a suitable support for heterogeneous catalysts [35,36,37]. In addition to being biocompatible, biodegradable, and low toxicity, its good antibacterial and antitumor properties enable chitosan to be applied for biomedical and pharmaceutical purposes [38,39]. Biocompatible heterogeneous catalysts could play an important role in chemical engineering [40,41]. M. Kamalzare et al., explored and considered magnetic bio-nanocomposite synthesized with chitosan and tannic acid that obtained successful advantages for use as a heterogeneous nanocatalyst [42]. In another study, they prepared a bio-nanocatalyst based on magnetic nanoparticles and starch as a natural polymer that lists mild condition, low cost, and non-toxicity as some of the advantages [43].

One of the important reactions catalysed by palladium is nitroarene reduction such as reductions of 4-nitrophenol (4-NP) and 4-nitroaniline (4-NA) [44,45]. The 4-NP is a toxic organic pollutant and can be converted to a less toxic 4-aminophenol (4-AP) using a simple reductive reaction catalysed by Pd NPs in the presence of NaBH_4_ as a reducing agent [46]. The 4-AP is a useful organic compound in pharmaceutical applications, such as the starting material for paracetamol synthesis [47]. The 4-NA is another nitroarene compound, which is classified as a type of hardly biodegradable organic pollutant commonly found in wastewater and associated with the dye synthesis industry [48]. It is toxic to aquatic organisms and harmful to human health after repeated exposure [49]. Therefore, the reduction of 4-NA to a more valuable and less toxic 4-phenylenediamine (4-PDA) is very important in many industries [50]. The 4-PDA produced from the reduction of the toxic 4-NA can be used as an ingredient in hair dyes [51], rubber antioxidants [52], aramid textile fibre intermediates, thermoplastics, and cast elastomers [53]. Besides nitroarene reduction, Pd can also play an important catalytic role in the allyl carbamate cleavage [54]. Due to the stability of allyl carbamate in acidic and basic conditions, it is widely used in the protection of the amine group that is crucial in certain organic synthesis reactions [55,56].

In this study, a novel chitosan-coated magnetic Pd NPs catalyst (Pd-Chit@MnFe_2_O_4_) was successfully designed for nitroarene reduction and allyl carbamate deprotection reactions. The magnetic material MnFe_2_O_4_ was coated with chitosan via the ionic gelation method to provide an ideal platform for metal ion immobilization on the support material. Subsequently, the immobilization of Pd NPs on the chitosan-coated MnFe_2_O_4_ was performed via the wet impregnation method. The physicochemical data and catalytic properties indicate that the catalyst has high potential for use in both chemical and biological systems.

## 2. Materials and Methods

### 2.1. Materials

Manganese (II) chloride tetrahydrate (≥98%), 1-amino-2-propanol (MIPA, 93%), chitosan (75–80% deacetylated, medium molecular weight ~190,000–310,000 Da), sodium tripolyphosphate (85%), palladium acetate (99.99%), 4-nitrophenol (≥98%), sodium borohydride (98%), rhodamine 110, allyl chloroformate, pyridine, and thiophenol were obtained from Sigma-Aldrich. 4-Nitroaniline, iron(III) chloride hexahydrate, and hydrochloric acid (37%) were purchased from Merck. Nitric acid (65%) was acquired from Friedemann Schmidt. A 10% hydrazine hydrate solution was prepared in methanol solvent based on the method reported by Liew et al. [12]. Fresh HeLa cells and MTT (3-(4,5-dimethylthiazol-2-yl)2,5-diphenyl tetrazolium bromide) were obtained from the Advanced Medical and Dental Institute (IPPT), Universiti Sains Malaysia (USM). All organic solvents were of analytical grade and used without further purification. Deionized water (DIW) was used throughout the experiments.

### 2.2. Preparation of Pd-Chit@MnFe_2_O_4_

#### 2.2.1. Preparation of MnFe_2_O_4_ Nanoparticles

MnFe_2_O_4_ nanoparticles were prepared using the coprecipitation method developed by Pereira et al. [57]. Solution A was prepared by mixing 10 mmol of MnCl_2_·4H_2_O with 5 mL of 0.1 M HCl (1:4 *V*/*V*) in a beaker. Solution B was prepared by dissolving 20 mmol of FeCl_3_·6H_2_O in 40 mL of DIW. Both solutions were heated to 50 °C and then quickly added to a beaker containing 200 mL of 3.0 M MIPA solution. The reaction mixture was heated to 100 °C and mechanically stirred for 2 h at 1000 rpm. The freshly prepared MnFe_2_O_4_ nanoparticles were magnetically isolated and dried under vacuum at room temperature overnight.

#### 2.2.2. Preparation of Chitosan-Coated MnFe_2_O_4_

Chitosan-coated MnFe_2_O_4_ was prepared based on the method developed by Liew et al. [58]. The mass ratio of MnFe_2_O_4_ to chitosan was 1:1.25. The concentration of chitosan solution was 5 mg·mL^−1^. The mass and volume ratio of chitosan to TPP used for ionic gelation was 5:1 and 5:2, respectively. The freshly prepared MnFe_2_O_4_ (100 mg) was dispersed in a beaker containing 25 mL of the acetic acid solution, followed by ultrasonication for 1 min at room temperature. Chitosan (125 mg) was added to the mixture and stirred at room temperature for 1 h at 1000 rpm. The TPP solution was prepared by dissolving 25 mg of TPP in 10 mL of DIW and then it was added dropwise to the MnFe_2_O_4_/chitosan mixture using a syringe pump at a rate of 1.0 mL·min^−1^. The mixture was then stirred at 1000 rpm for 1 h at room temperature. The resulting product, Chit@MnFe_2_O_4_ was separated using a magnet, washed with water until pH = 7, and dried under vacuum at room temperature overnight.

#### 2.2.3. Preparation of Magnetic-Supported Pd Catalyst

Magnetic-supported Pd catalyst Pd-Chit@MnFe_2_O_4_ was prepared by adding 100 mg of Chit@MnFe_2_O_4_ in a beaker containing 5 mL of toluene and 0.05 mmol of Pd(OAc)_2_ salt. The reaction mixture was stirred at 80 °C at 40 rpm for 10 min and then further stirred at room temperature for 2 h. Subsequently, the Pd^2+^ ion was reduced to Pd NPs by adding 10% hydrazine hydrate solution and stirred for 30 min at room temperature with a stirring speed of 40 rpm. The freshly prepared Pd-Chit@MnFe_2_O_4_ was then separated, washed with acetone, and dried under vacuum for 24 h. Superparamagnetic MnFe_2_O_4_ was first synthesized through the coprecipitation method by using MIPA as the alkaline agent. Subsequently, the superparamagnetic MnFe_2_O_4_ was coated with chitosan via the ionic gelation method using sodium tripolyphosphate (TPP) as a cross-linking agent. The coating approach resulted in tuned surface properties of MnFe_2_O_4_, which are ideal for use as a support for heterogeneous catalysts because chitosan has a high sorption capacity for metals and metal ions [59]. For Pd NPs immobilization, Pd(OAc)_2_ was used as a Pd precursor to the introduction of free palladium(II) ions in toluene. The free palladium(II) ions were physically adsorbed on the surface of the Chit@MnFe_2_O_4_ via electrostatic interaction. The solid material was then treated with hydrazine hydrate to reduce the palladium(II) ions to metallic palladium nanoparticles (Pd NPs). The loading of Pd on Pd-Chit@MnFe_2_O_4_ was further investigated by ICP-MS.

#### 2.2.4. Preparation of Rhodamine 110

**Scheme 1:**
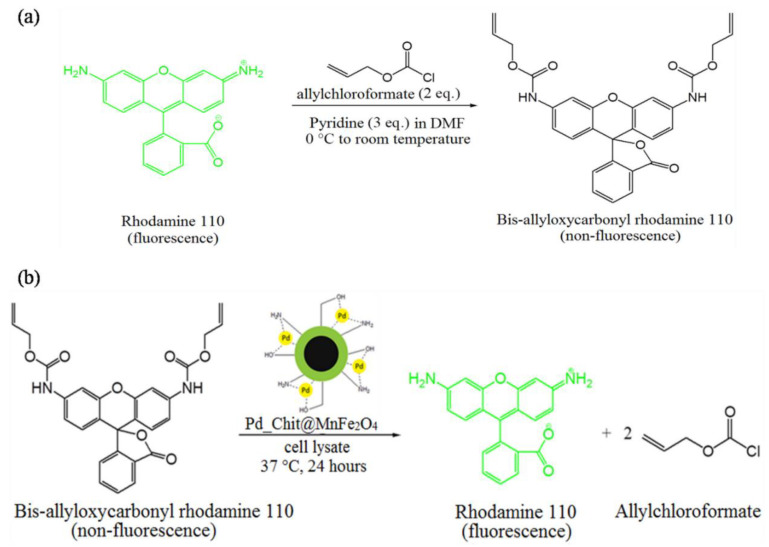
(**a**) Synthesized of non-fluorescence bis-allyloxycarbonyl protected rhodamine 110 and (**b**) Palladium-induced allyl carbamate cleavage by Pd-Chit@MnFe_2_O_4_ in HeLa cell lysate to produce fluorescence rhodamine 110. The product of the reaction was fluorescence rhodamine 110. This reaction was important to confirm the catalytic performance of Pd-Chit@MnFe_2_O_4_ was not inhibited by components present in cells.

Bis-allyloxycarbonyl-protected rhodamine 110 was produced based on the method published by Streu and Meggers [40]. Rhodamine 110 (0.5 mmol) was dissolved in 1.2 mL of dry *N*,*N*′-dimethylformamide (DMF). The reaction mixture was cooled down to 0 °C under an inert atmosphere. A mixture of allyl chloroformate (1.0 mmol) and pyridine (1.5 mmol) in DMF (0.5 mL) was prepared and then added dropwise to the previous solution containing rhodamine 110. The reaction mixture was warmed to room temperature and stirred for 24 h. The product was extracted using ethyl acetate and concentrated under a vacuum. Column chromatography technique was performed by using hexane: ethyl acetate (2:1) to obtain the pure product. The preparation of non-fluorescence bis-allyloxycarbonyl-protected rhodamine 110 from a fluorescence compound rhodamine 110 by using allyl chloroformate is shown in Scheme 1.

### 2.3. Catalyst Characterization

#### 2.3.1. Structural Analysis

The structure changes on the synthesized sample were investigated using X-ray diffraction (XRD, Bruker D8-Advanced (Bruker AXS, Bremen, Germany). The instrument employed Cu-Kα radiation (λ = 0.15406 nm) at 30 kV and 15 mA, and scans were conducted over the 2θ range of 20° ≤ 2θ ≤ 80° with a scanning rate of 2° min^−1^. The patterns were matched to the Joint Committee of Powder Diffraction Standard (JCPDS)’s database. The average crystallite size of Pd and MnFe_2_O_4_ was calculated by using the Debye–Scherrer formula [60]. The elemental composition and chemical state of the catalyst were determined using X-ray photoelectron spectrometry (XPS) analysis (Kratos Axis Ultra DLD X-ray photoelectron spectrometer). The catalyst sample was prepared in a pellet formed for XPS analysis. The determination of the functional group present on the prepared catalyst was investigated using Fourier transform infrared (FTIR) spectra using Agilent Technologies Cary 630 FTIR spectrometer in the range of 650–4000 cm^−1^. Furthermore, the morphology and particle size of the catalyst was examined by transmission electron microscopy (TEM) using Philips CM 12 transmission electron microscope at 100 kV and field emission scanning electron microscopy (FESEM) using SUPRA 55VP Zeiss scanning electron microscope.

#### 2.3.2. Elemental Analysis

The elemental composition of the Pd immobilized on the surface of Chit@MnFe_2_O_4_ was determined by inductively coupled plasma mass spectrometry (ICP-MS) using a Perkin Elmer ELAN 9000 ICP mass spectrometer (USA). The loading of Pd immobilized on the surface of Chit@MnFe_2_O_4_ was determined by dissolving 1.0 mg of Pd-Chit@MnFe_2_O_4_ in 3 mL aqua regia. The solution was then topped-up with water to a total volume of 25 mL. The amount of Pd in the solution was determined by ICP-MS.

#### 2.3.3. Magnetic Properties

The magnetic properties of the sample were analysed using vibrating sample magnetometry (VSM) (LakeShore 7404 series) vibration sample magnetometer. To distinguish the metal-oxygen species in the catalyst precursors, UV-Vis measurements were performed using a Shimadzu UV-2450 UV-Vis spectrophotometer over a wavelength range of 200~800 nm.

#### 2.3.4. Catalytic Activity

##### Catalytic Reduction of Nitroarene Compounds and Reusability

The catalytic activity of Pd-Chit@MnFe_2_O_4_ was investigated in nitroarene (4-NP and 4-NA) reduction using NaBH_4_ as a reducing agent. In a typical reaction, 0.15 mmol of NaBH_4_ was added to 3 mL of 0.05 mM 4-NP solution, resulting in a yellow-green solution of 4-nitrophenolate. The Pd-Chit@MnFe_2_O_4_ was then added to the solution mixture and the conversion of 4-NP to 4-AP took place immediately. The reduction reaction was monitored using a UV-Vis spectrometer. The optimization of 4-NP reduction was performed with the same procedures by using different masses (0.5 and 1.0 mg) of Pd-Chit@MnFe_2_O_4_. The reduction of 4-NA to 4-PDA was also carried out with the same experimental conditions by using 0.5 mg of Pd-Chit@MnFe_2_O_4_ as the catalyst. Control experiments were carried out for both 4-NP and 4-NA reduction respectively by replacing the catalyst with MnFe_2_O_4_, Chit@MnFe_2_O_4_, and without catalyst. The rate of reaction was calculated from the pseudo-first-order kinetic model:

(1) $$\ln(A/A_{0})=-kt$$

where *A* is the concentration of nitroarene compound at *t* time, *A*_0_ is the initial concentration of 4-NP or 4-NA and *k* is the rate of the reaction [28]. For a more accurate comparison, the turnover frequency (TOF) value was calculated according to the equation:

(2) $$TOF=n_{0}/(n_{pd}\times reaction\;time)$$

where 𝑛_0_ is the initial amount of 4-NP or 4-NA and 𝑛_Pd_ is the number of Pd active sites on the catalyst used. The reusability of Pd-Chit@MnFe_2_O_4_ was tested using 20 times scale-up nitroarene reduction in a 50 mL round bottom flask with the same reaction conditions. The catalyst was isolated using a magnet, washed with water, and used in the next cycle of nitroarene reduction. The mass of the isolated catalyst was weighed after all 8 cycles of nitroarene reduction were completed.

##### Deprotection of Bis-allyloxycarbonyl Rhodamine 110 with and without Thiophenol

Deprotection of bis-allyloxycarbonyl rhodamine 110 was performed according to the method developed by Yusop et al. [41]. Deprotection of bis-allyloxycarbonyl rhodamine 110 was carried out in two conditions, with thiophenol, and without thiophenol. For the condition with thiophenol, 1.5 µmol of bis-allyloxycarbonyl-protected rhodamine 110 in dimethyl sulfoxide (DMSO) (75 µL, 20 mM) was added to 2.72 mL of the cell lysate solution, followed by the addition of 150 µL of a 100 mM thiophenol solution in DMSO. Then, 60 µL of Pd-Chit@MnFe_2_O_4_ in DIW (10 mM, Pd loading of 0.6 µmol) was added to the mixture. The final reaction mixture (3 mL) was shaken at 300 rpm at 37 °C for 24 h. The reaction was monitored by thin-layer chromatography (TLC) and observed under UV light. For the reaction without thiophenol, 1.5 µmol of bis-allyloxycarbonyl-protected rhodamine 110 in DMSO (75 µL, 20 mM) was added to 2.87 mL of cell lysate solution, followed by the same amount of Pd-Chit@MnFe_2_O_4_. A negative control experiment also was carried out without adding Pd-Chit@MnFe_2_O_4_ and thiophenol for comparison. Negative control was performed by adding 2.93 mL of the cell lysate solution to 1.5 µmol of bis-allyloxycarbonyl-protected rhodamine 110 in DMSO (75 µL, 20 mM).

##### Stability and Cytotoxicity of Pd-Chit@MnFe_2_O_4_

The stability of Pd-Chit@MnFe_2_O_4_ was investigated by robustness test. Approximately 1.0 mg of Pd-Chit@MnFe_2_O_4_ was dispersed in 10 mL of DIW and heated to 80 °C for 20 min with a stirring speed of 40 rpm. The Pd-Chit@MnFe_2_O_4_ was filtered off and the filtrate was examined by ICP-MS. The robustness test for Pd on manganese ferrite without coating was carried out using the same procedure as Pd-Chit@MnFe_2_O_4_. A cytotoxicity test was carried out on the HeLa cell by using an MTT assay with two concentrations of Pd-Chit@MnFe_2_O_4_, which were 1.25 × 10^−1^ mg·mL^−1^ and 2.5 × 10^−1^ mg·mL^−1^ of Pd-Chit@MnFe_2_O_4_ in DIW. A cell density of 5000 cells/well was plated in a 96-well plate and allowed to grow for 24 h. Then, the Pd-Chit@MnFe_2_O_4_ was added to the well-incubated HeLa cell and monitored with a fluorescence microscope. After 24 h, the cytotoxicity test result was compared with the untreated cell. The cytotoxicity test was performed in triplicate for both concentrations of Pd-Chit@MnFe_2_O_4_.

## 3. Results and Discussion

### 3.1. Structural Analysis

**Figure 1:**
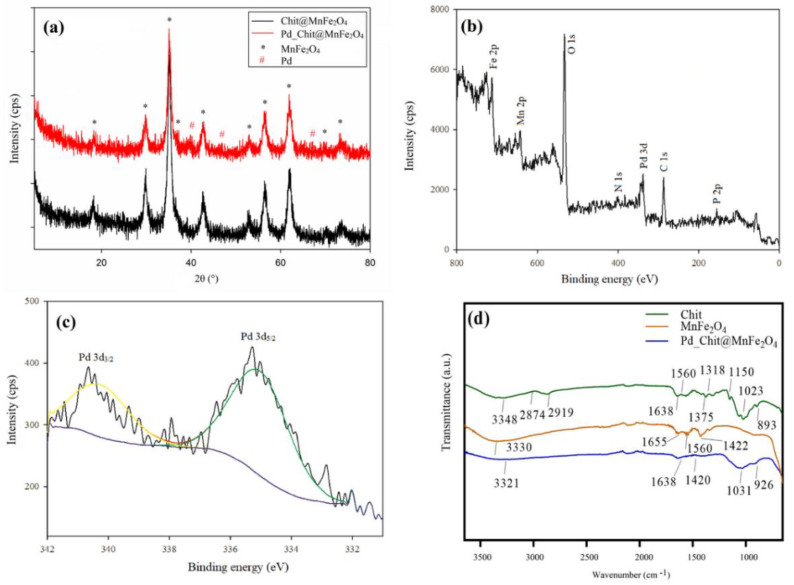
(**a**) XRD patterns, (**b**) wide scan XPS spectrum, (**c**) Pd 3*d* high resolution XPS spectrum and (**d**) FTIR spectrum of Pd-Chit@MnFe_2_O_4_.

The XRD diffractogram of Chit@MnFe_2_O_4_ and Pd-Chit@MnFe_2_O_4_ are shown in Figure 1a. The result showed there are ten diffraction peaks related to the MnFe_2_O_4_ with spinel cubic crystal can be observed at 2*θ* = 18.5°, 30.0°, 35.5°, 37.0°, 42.5°, 53.0°, 56.5°, 62.0°, 70.0°, and 73.5°. These 2*θ* correspond to the (111), (220), (311), (222), (400), (422), (511), (440), (620), and (533) planes of the MnFe_2_O_4_ crystal’s unit cell (JCPDS 01-073-3820), respectively [61,62]. From the XRD diffractogram of Pd-Chit@MnFe_2_O_4_, three weak diffraction peaks were observed at 2*θ* = 40.1°, 46.6°, and 68.3°, which were assigned to the (111), (200), and (220) planes of Pd(0), respectively, with a face-centred cubic lattice structure (JCPDS 00-046-1043) [63]. The weak intensity of the diffraction peaks is due to the low Pd content on the catalyst surface. However, the intensity of diffraction peaks related to MnFe_2_O_4_ reduced due to chitosan coating and immobilization of Pd NPs [64]. The mean crystallite size of MnFe_2_O_4_ was calculated using the Scherrer equation was determined to be 11.9 nm whereas the mean crystallite size of Pd was around 3.4 nm. Noteworthy to mention, the diffraction peaks related to chitosan were not observed in the XRD diffractogram of Chit@MnFe_2_O_4_ and Pd-Chit@MnFe_2_O_4_ due to the amorphous nature of chitosan or due to the formation of a thin chitosan layer [65]. The presence of diffraction peaks related to MnFe_2_O_4_ in the diffractogram of Chit@MnFe_2_O_4_ and Pd-Chit@MnFe_2_O_4_ indicates that its crystallinity is preserved even in the presence of chitosan and Pd.

The XPS spectrum of Pd 3*d* is given in Figure 1b–c. The deconvolution of the spectrum resulted in two peaks centred at *BE* = 334.8 eV and *BE* = 340.8 eV. The first peak is attributed to the Pd 3*d*_5/2_ whereas the latter is attributed to the Pd 3*d*_3/2_, which can be ascribed to metallic Pd. The presence of these peaks confirmed that Pd^2+^ was mainly reduced to metallic Pd. The XPS result is consistent with the previously reported reports [66]. The minor peaks located at 338.2 eV (Pd 3*d*_5/2_) and 343.4 eV (Pd 3*d*_3/2_) correspond to Pd(II) species, which have indicated the interaction of metallic Pd with amine groups in chitosan [67,68].

**Figure 2:**
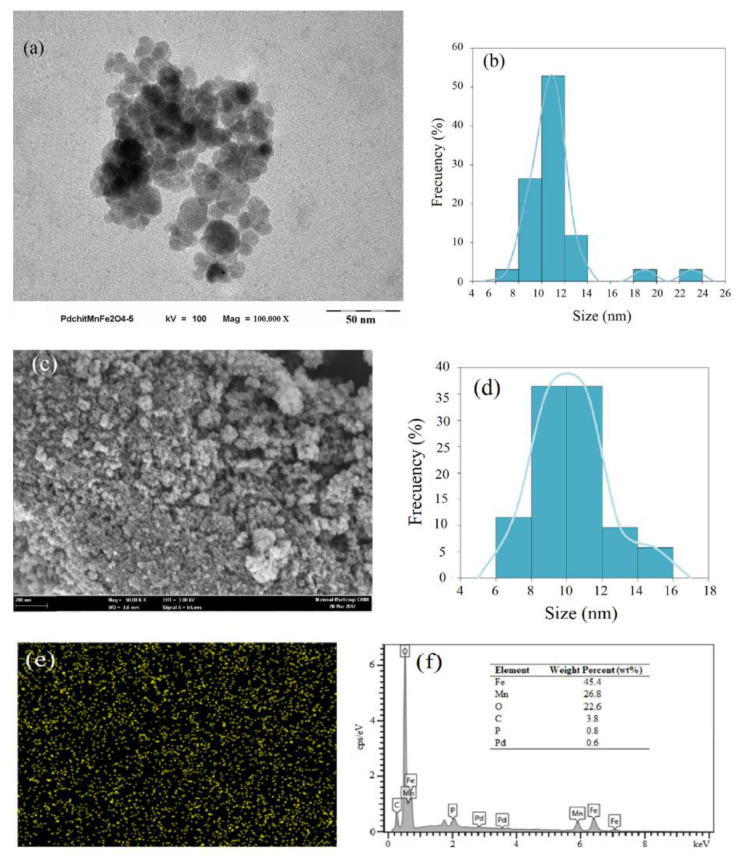
(**a**) TEM micrograph of Pd-Chit@MnFe_2_O_4_, (**b**) the size distribution histogram from TEM micrograph, (**c**) TEM micrograph of Pd, (**d**) the size distribution histogram from TEM micrograph, (**e**) SEM micrograph of Pd-Chit@MnFe_2_O_4_, (**f**) the size distribution histogram from SEM micrograph, (**g**) mapping micrograph for Pd NPs, and (**h**) EDS data of Pd-Chit@MnFe_2_O_4_.

Figure 1d shows the FTIR spectrum of chitosan, MnFe_2_O_4_, and Pd-Chit@MnFe_2_O_4_. In the FTIR spectrum of chitosan, the absorption band showed at 3348 cm^−1^ (–O–H) [69], 2874 cm^−1^ and 2919 cm^−1^ (–CH_2_ and –CH_3_) [70], 1638 cm^−1^, 1560 cm^−1^, and 1318 cm^−1^ (–C=O, stretching of amide I, –N–H bending of amide II, and –C–N stretching of amide III) [71], 1375 cm^−1^ (–C–H) [72], 893 cm^−1^ and 1150 cm^−1^ (C–O–C stretching of glycosidic linkages) [73], 1060 cm^−1^ and 1023 cm^−1^ (–C–O stretching vibration of a secondary alcohol and primary alcohol), respectively [74]. A similar trend was observed on Pd-Chit@MnFe_2_O_4_, yet Pd-Chit@MnFe_2_O_4_ showed shifting of the stretching vibrations of chitosan’s (–C–O–C) from 1023 to 1031 cm^−1^. The shifting indicates that the bond strengths of C–O–C in glycosidic bonds increased due to environmental differences after the ionic gelation process [75]. Noted, it also implied that manganese ferrite was successfully coated with chitosan via cross-linked TPP. The case of MnFe_2_O_3_ showed the elimination of absorption peaks belonging to –CH_2_, –CH_3,_ and C–O–C. The morphology and particle size distributions of Chit@MnFe_2_O_4_ and of Pd-Chit@MnFe_2_O_4_ were characterized by SEM and TEM analyses, respectively. From Figure 2a,c, it can be observed that the MnFe_2_O_4_ particles were mostly spherical-shaped with various sizes. The surface morphology of the catalyst was spherically shaped as well (Figure 2e). The average diameter of Pd-Chit@MnFe_2_O_4_ determined from the TEM image was about 11 ± 5 nm (Figure 2b) whereas the average diameter of Pd-Chit@MnFe_2_O_4_ determined from the SEM image was 10 ± 2 nm (Figure 2f). Figure 2c is the TEM micrograph of Chit@MnFe_2_O_4_ showing the average particle size from the corresponding diameter distribution, which is in the range of 6 nm (Figure 2d). Thus, the reported higher size in Pd-Chit@MnFe_2_O_4_ is due to the capping of Pd nanoparticles. The catalytic activity of the Pd catalyst is probably due to the following effects: A high absorption capacity was provided by the morphology of Pd nanocomposite which in turn, decreased the induction time. Additionally, the catalytic activity was improved due to the strong synergistic effects of its constituents. (−NO_2_) group can be reduced to an amine and aniline by the Pd-Chit@MnFe_2_O_4_ nanocomposite. Without Pd as catalytic hydrogenation, the catalytic reduction reaction cannot proceed. As the catalyst was added into the system, ions were adsorbed by Pd, due to strong adsorption capacity and reduction reaction removed oxygen and added hydrogen. Then the active species reduced −NO_2_ into −NH_2_, which indicated the catalyst’s critical role in this reduction. In Figure 2h, EDS analysis indicates that Pd-Chit@MnFe_2_O_4_ was mainly composed of C, O, Fe, and Mn, P, and Pd. The P originated from the CS cross-linked with the TPP ion by ionic gelation [58]. The EDS and mapping micrograph proved that the immobilized Pd NPs were well-dispersed on the Chit@MnFe_2_O_4_ support (Figure 2g).

### 3.2. Elemental Analysis

The total concentration of Pd, Mn, and Fe in Pd-Chit@MnFe_2_O_4_ detected by ICP-MS analysis is given in Table 1. The loading of Pd on Pd-Chit@MnFe_2_O_4_ was determined to be 0.21 mmol·g^−1^. The atomic ratio of Fe: Mn was determined to be 2:1, which agrees with the molecular formula of MnFe_2_O_4_. The robustness test indicates that higher Pd leached out from the MnFe_2_O_4_ without chitosan coating. The concentration of Pd detected was 26.6 ppb or 0.03 wt%. The Pd concentration was reduced to 10.8 ppb or 0.01 wt% when the MnFe_2_O_4_ was coated with chitosan. The observation indicates that the Pd-Chit@MnFe_2_O_4_ is safe to be used in the pharmaceutical industry as the leaching level of Pd from the catalyst was lower than the maximum acceptable concentration limits [76,77].

### 3.3. Magnetic Properties

**Figure 3:**
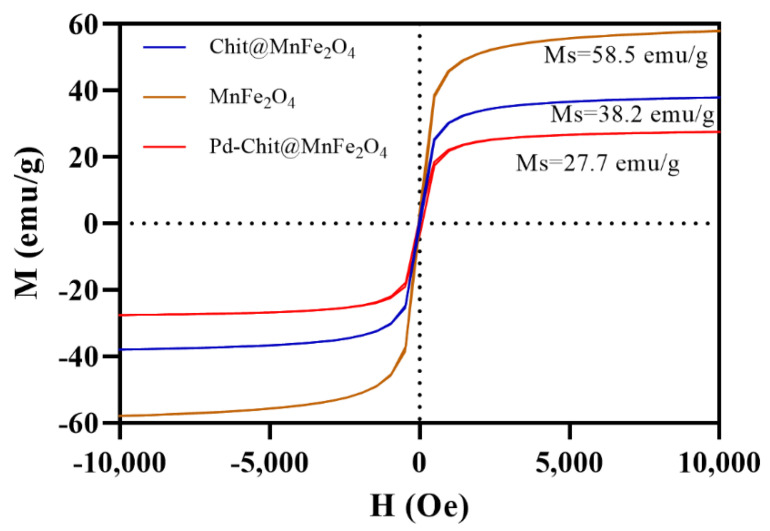
Magnetization curves of MnFe_2_O_4_, Chit@MnFe_2_O_4,_ and Pd-Chit@MnFe_2_O_4_.

The magnetic properties of Pd-Chit@MnFe_2_O_4_ were observed using VSM at room temperature and the magnetization curves are shown in Figure 3 and Table 2. The MnFe_2_O_4_, Chit@MnFe_2_O_4_, and Pd-Chit@MnFe_2_O_4_ retained high saturation magnetization (M_s_) with low coercivity (H_c_) and negligible remanent magnetization (M_r_). The low squareness ratio (M_r_/M_s_) of MnFe_2_O_4_ (0.03), Chit@MnFe_2_O_4_ (0.03), and Pd-Chit@MnFe_2_O_4_ (0.05) confirmed their superparamagnetic behaviour [78]. Of note, the M_s_ value of MnFe_2_O_4_ was slightly higher than that of Chit@MnFe_2_O_4_ because of the chitosan coating around MnFe_2_O_4_ [79], whereas the decrease in the M_s_ value of Pd-Chit@MnFe_2_O_4_ indicated that Pd was successfully immobilized on Chit@MnFe_2_O_4_ [80]. The coating of chitosan and immobilization of Pd form a magnetically disordered layer around MnFe_2_O_4_, which results in a decrease in the total amount of magnetic phase of the support material and catalyst. The superparamagnetic properties of Pd-Chit@MnFe_2_O_4_ allowed the catalyst to be easily separated and recycled by applying an external magnetic field; it was redispersed when the external magnetic field was removed.

### 3.4. Catalytic Activity

#### 3.4.1. Catalytic Reduction of Nitroarenes and Reusability

**Figure 4:**
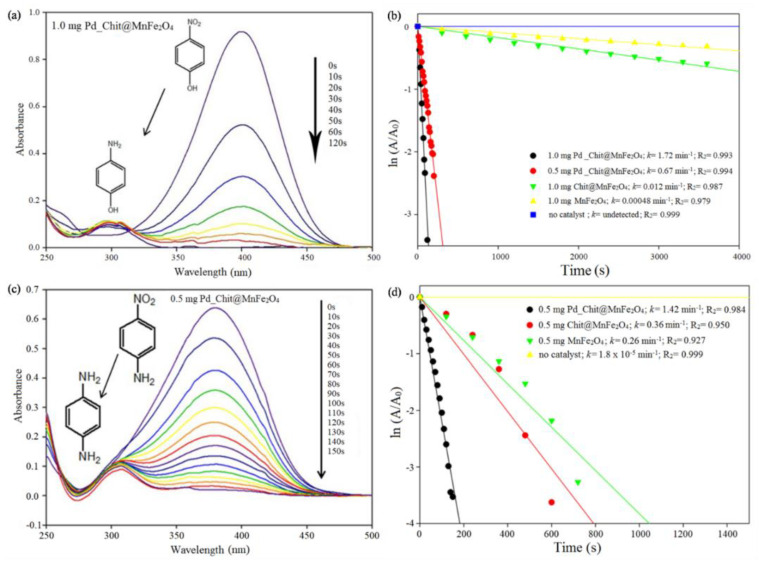
4−NP reduction’s (**a**) UV−Vis spectra with 1.0 mg Pd-Chit@MnFe_2_O_4_ and (**b**) pseudo-first-order kinetic plot (**c**) UV−Vis spectra with 0.5 mg Pd-Chit@MnFe_2_O_4_ and (**d**) pseudo-first-order kinetic plot.

The catalytic performance of Pd-Chit@MnFe_2_O_4_ was investigated in the reduction of 4−NP and 4-NA using NaBH_4_ as a reducing agent. Due to the excess NaBH_4_, the reaction rate depends only on the concentration of the nitroarene compound. Hence, a pseudo-first-order kinetic plot was used to study the rate constant of the nitroarene reduction (Figure 4b,d). The 4−NP reduction profile is shown in Figure 4a. The absorption band at 400 nm belongs to the 4-nitrophenolate ion, which was formed when NaBH_4_ was added to the 4−NP solution [81]. The absorption band continuously decreased in the presence of the catalyst. The reduction of the 4-nitrophenolate ion is accompanied by the formation of a new absorption band around 300 nm, which indicates the formation of 4−AP [82].

The effect of catalyst type and the effect of Pd-Chit@MnFe_2_O_4_ mass on the 4-NP reduction reaction was also investigated. The pseudo-first-order plot of the 4-NP reduction is shown in Figure 4b, whereas the *k* and TOF values are summarized in Table 3. The catalytic reduction of 4-NP was low when Chit@MnFe_2_O_4_ and MnFe_2_O_4_ were applied as catalysts. Upon the addition of 0.5 mg of Pd-Chit@MnFe_2_O_4_, the reduction reaction of 4-NP requires 200 s to complete with a rate of reaction of 0.67 min^−1^ and a TOF of 285.7 min^−1^. The increase in the rate of catalytic reduction of 4-NP confirmed that Pd plays an important role in the reduction of 4-NP. When the mass of Pd-Chit@MnFe_2_O_4_ increased to 1.0 mg, the reaction time was reduced to 120 s. The *k* and TOF values for 1.0 mg of Pd-Chit@MnFe_2_O_4_ were 1.72 min^−1^ and 357.1 min^−1^, respectively.

The catalytic activity of Pd-Chit@MnFe_2_O_4_ was further evaluated in the reduction of 4-NA. From Figure 4c, the intensity of an absorption band around 380 nm associated with 4-NA decreased as the reaction continued [83]. The reduction in the absorption intensity of 4-NA is accompanied by the rise in an absorption band associated with 4-PDA around 305 nm [40]. For the control experiment, the pseudo-first-order plots of 4-NA reduction are illustrated in Figure 4d, whereas the *k* and TOF values are shown in Table 3. In the control experiments, the reaction rate was slow when the experiment was conducted with 0.5 mg of Chit@MnFe_2_O_4_ and MnFe_2_O_4_. The conversion of 4-NA to 4-PDA was negligible without a catalyst. Noteworthy, when 0.5 mg Pd-Chit@MnFe_2_O_4_ was added, the reduction of 4-NA was catalysed at a higher reaction rate of 1.42 min^−1^ and TOF value of 571.4 min^−1^. The complete reaction time was reduced to 2.5 min. Overall, the results showed that Pd-Chit@MnFe_2_O_4_ has superior catalytic activity in a reduction reaction of 4-NP and 4-NA.

**Figure 5:**
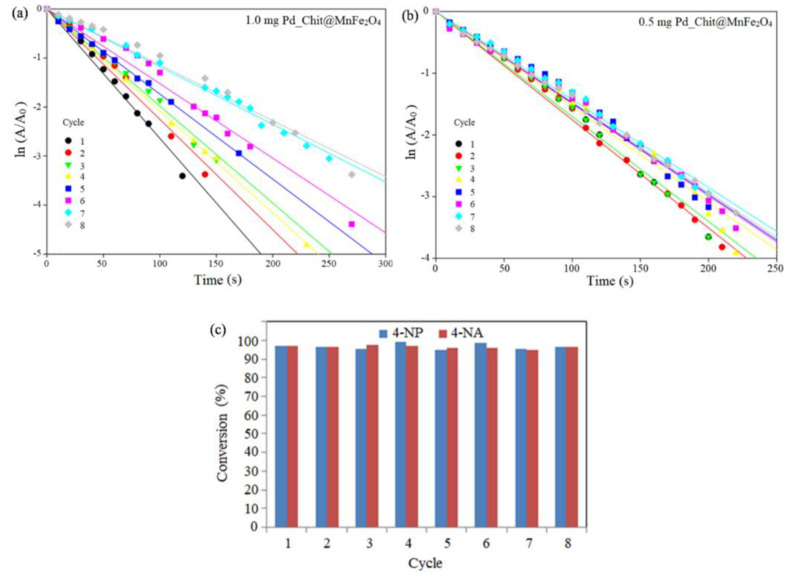
Reusability of Pd-Chit@MnFe_2_O_4_ in(**a**) 4-NP and (**b**) 4-NA reductions and (**c**) conversion of 4-NP and 4-NA reduction.

The pseudo-first-order kinetic plot of 8 consecutive cycles of 4-NP and 4-NA reduction reactions is displayed in Figure 5a and Figure 5b, respectively. Results show that the conversion of 4-NP and 4-NA was ~100% for 8 consecutive cycles (as shown in Figure 5c), which indicated that Pd-Chit@MnFe_2_O_4_ was stable and can be reused up to 8 consecutive cycles. The minimum loss of catalyst nanoparticles during the recycling process may lead to a slight increase in reaction time upon cycles for both 4-NP and 4-NA reduction.

**Figure 6:**
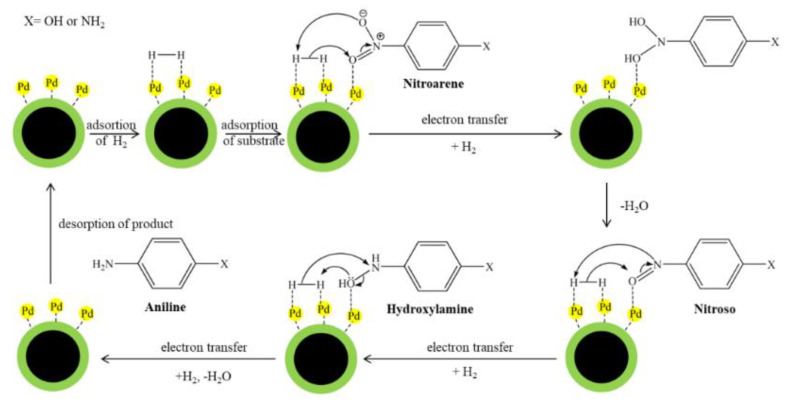
The proposed mechanism for Pd-Chit@MnFe_2_O_4_ catalyzed nitroarenes reduction, which involved adsorption of reactants, transfer of electrons, and adsorption of the product occurred at the surface of the catalyst.

A plausible mechanism was proposed for Pd-Chit@MnFe_2_O_4_ catalysed nitroarenes reduction based on the Langmuir–Hinshelwood model and Haber mechanism [84,85]. The proposed mechanism is shown in Figure 6. First, both reactants adsorbed on the surface-active site of Pd-Chit@MnFe_2_O_4_ based on the Langmuir–Hinshelwood model. After the adsorption process, electron transfer occurred between the nitroarene compound and hydride ion, followed by the dehydration process to yield the nitroso compound. The nitroso compound undergoes further reduction to form hydroxylamine compound in a very fast step. Next, the hydroxylamine compound was finally converted to an amine compound via a series of electron transfers followed by the dehydration process. At the end of the catalytic cycle, the amine compound as product desorbed from the surface of Pd-Chit@MnFe_2_O_4_, and a new catalytic cycle will begin.

#### 3.4.2. Palladium-Induced Allyl Carbamate Deprotection

**Figure 7:**
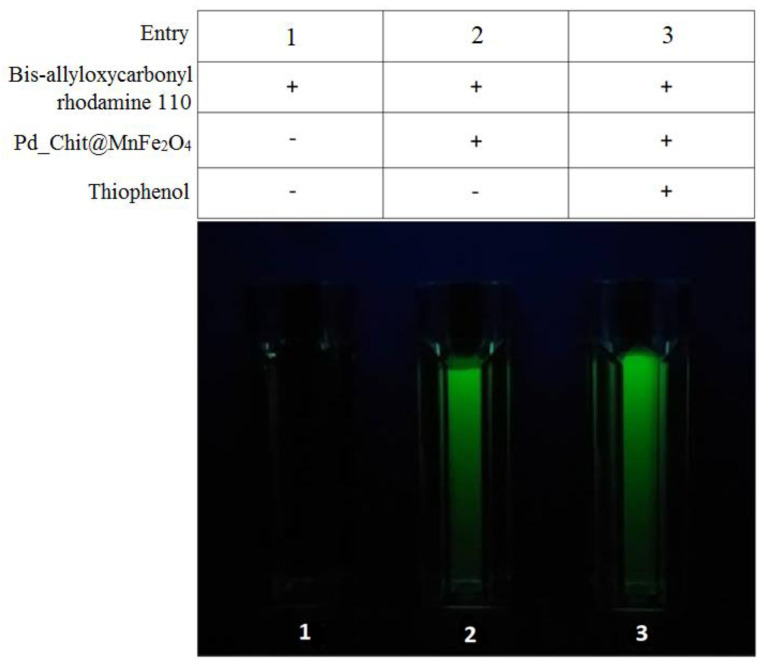
Deprotection of bis-allyloxycarbonyl rhodamine 110 in 3 different conditions. Reaction conditions: Entry 1: negative control using bis-allyloxycarbonyl rhodamine 110 in DMSO (75 µL, 20 mM) and cell lysate solution (2.93 mL); Entry 2: bis-allyloxycarbonyl rhodamine 110 in DMSO (75 µL, 20 mM), cell lysate solution (2.87 mL) and Pd-Chit@MnFe_2_O_4_ in water (60 µL, 10 mM); Entry 3: bis-allyloxycarbonyl rhodamine 110 in DMSO (75 µL, 20 mM), cell lysate solution (2.72 mL), Pd-Chit@MnFe_2_O_4_ in water (60 µL, 10 mM), and thiophenol in DMSO (150 µL, 100 mM). All reactions were shaken at 300 rpm for 24 h at 37 °C.

From Figure 7 (entry 1), the control experiment showed that no product was formed (no fluorescence detected) in the absence of Pd-Chit@MnFe_2_O_4_, whereas the deprotection of bis-allyloxycarbonyl rhodamine 110 was successfully performed in the presence of Pd-Chit@MnFe_2_O_4_ with and without thiophenol (Figure 7: entry 3). Notably, our newly designed catalyst Pd-Chit@MnFe_2_O_4_ successfully catalysed allyl carbamate cleavage without thiophenol as a scavenger (Figure 7: entry 2). High levels of thiophenol have proven to be toxic to living organisms and the environment; therefore, this approach without thiophenol is highly beneficial [86]. The characterization of the reaction products formed after the reaction was determined by the purification and analysis of the cell lysate, which quantitatively confirmed the chemical identities via liquid chromatography–mass spectrometry (LC-MS) and high-performance liquid chromatography (HPLC). The reaction mixture was centrifuged for 5 min at 13,000 rpm. The supernatant was collected and passed through a DSC-18LT column which had been prewashed with water. The column was washed with water to remove salts and proteins from the cell lysate, and acetonitrile was used to elute the desired product from the column. The residue was dissolved in methanol and analysed by LC-MS in a positive ionization mode. LC-MS suggested the presence of rhodamine 110 with the m/z found at 331.0. In addition, HPLC analysis of the cell lysate showed retention times with a sample standard of around 3.2 min.

### 3.5. Cytotoxic Assay

**Figure 8:**
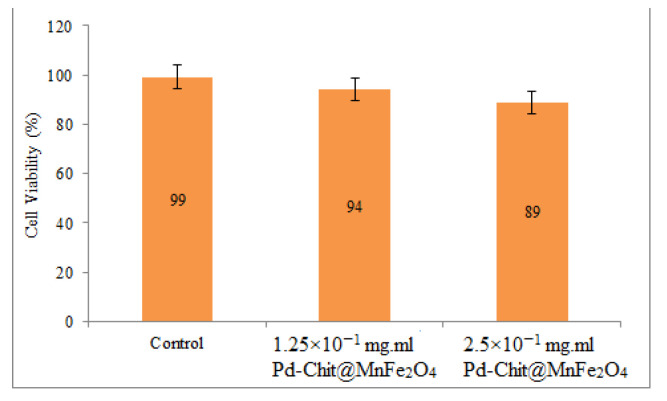
Cytotoxicity of Pd-Chit@MnFe_2_O_4_ on HeLa cells at 24 h.

A HeLa cell was chosen to investigate the cytotoxicity of Pd-Chit@MnFe_2_O_4_ because of the cell’s ability to grow rapidly and easily [87]. Figure 8 depicts the cytotoxic evaluation of Pd-Chit@MnFe_2_O_4_. As can be seen, Pd-Chit@MnFe_2_O_4_ caused low necrosis of HeLa cells and high cell viability (>89%) was observed after 24 h. The viability of HeLa cells treated with Pd-Chit@MnFe_2_O_4_ at two concentrations was found to have no substantial cytotoxicity. Previous literature has reported on low cytotoxicity heterogeneous Pd catalyst catalysing Suzuki–Miyaura cross-coupling and allyl carbamate cleavage in vivo [41]. Due to the low cytotoxicity of Pd-Chit@MnFe_2_O_4_, the Pd-Chit@MnFe_2_O_4_ catalyst is suggested as a potential candidate for catalysis in living systems. 

### 3.6. BET Analysis

**Figure 9:**
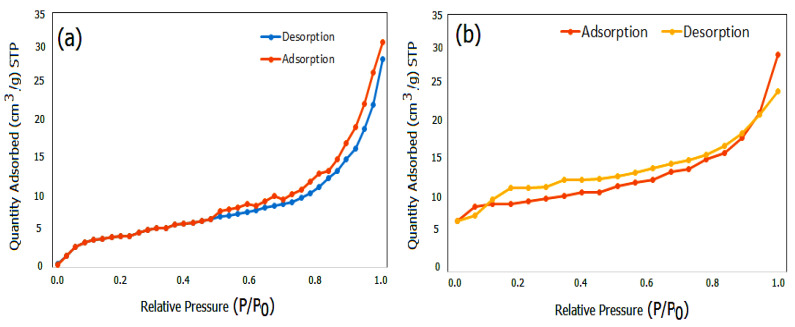
BET surface area analysis of (**a**) palladium nanoparticles, (**b**) Pd-Chit@MnFe_2_O_4_.

The surface area of the pure Pd and Pd-Chit@MnFe_2_O_4_ samples was estimated by Brunauer–Emmett–Teller surface area analysis (BET) (Figure 9a and Figure 9b, respectively). According to the results, the surface area of 25.614 and 2.292 m^2^·g^−1^ were found for palladium nanoparticles. The higher BET specific surface area of the Pd-Chit@MnFe_2_O_4_ nanocomposite compared with pure Pd can be ascribed to the introduction of Chit@MnFe_2_O_4_ particles in the composite and is beneficial to improve the catalytic performance.

## 4. Conclusions

Chit@MnFe_2_O_4_ was successfully prepared and used as supporting material for heterogeneous Pd NP catalysts. This newly prepared Pd-Chit@MnFe_2_O_4_ catalyst demonstrated high catalytic activities in nitroarene reduction as well as excellent catalytic performance in the deprotection of allyl carbamate under biological conditions. Moreover, Pd-Chit@MnFe_2_O_4_ was stable for reuse and could be recycled with negligible loss of catalytic activity. The recovery process of Pd-Chit@MnFe_2_O_4_ was easy and convenient due to its superparamagnetic properties. Furthermore, this magnetically active chitosan-coated Pd catalyst is biocompatible and eco-friendly because it exhibited low cytotoxicity and minimal leaching problems. Thus, it could be suitable for a variety of chemical applications. The biocompatible Pd-Chit@MnFe_2_O_4_ is of interest for future applications, predominantly in biomedical and clinical research.

## Figures and Tables

**Table 1:** ICP-MS results for Pd-Chit@MnFe_2_O_4_. *1.0 mg Pd-Chit@MnFe_2_O_4_ was dissolved in 3 mL aqua regia and top-up with water to a total volume of 25 mL.

Analyte	Mean Concentration (ppb)	Metal Loading (mmol/g)	Simplest Ratio
Palladium (Pd)	912.75	0.21	-
Manganese (Mn)	7286.01	3.32	1
Iron (Fe)	13,523.70	6.05	1.8

**Table 2:** Magnetic properties of MnFe_2_O_4_, Chit@MnFe_2_O_4_, and Pd-Chit@MnFe_2_O_4_.

Nanomaterial	Magnetic Characterization			
	Ms (emu/g)	H_c_ (O_e_)	Mr (emu/g)	M_r_/M_s_
MnFe_2_O_4_	58.4	22.8	2.0	0.03
Chit@MnFe_2_O_4_	38.2	22.5	1.2	0.03
Pd-Chit@MnFe_2_O_4_	27.7	31.4	1.3	0.05

**Table 3:** The chemical equation and comparison of catalytic performance of Pd-Chit@MnFe_2_O_4_ for the 4-NP and 4-NA reduction in various experimental conditions.

The chemical equation of nitroarene reduction: 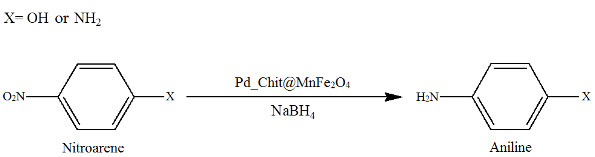			
**Condition**	**Time (s)**	**k (min^−1^)**	**TOF (min^−1^)**
(a) 4-NP reduction *:			
1.0 mg Pd-Chit@MnFe_2_O_4_	120	1.72	357.1
0.5 mg Pd−Chit@MnFe_2_O_4_	200	0.67	285.7
1.0 mg Chit@MnFe_2_O_4_	3600	0.012	-
1.0 mg MnFe_2_O_4_	3600	0.0048	-
Without catalyst	3 days	-	-
(b) 4-NA reduction *:			
0.5 mg Pd-Chit@MnFe_2_O_4_	150	1.42	571.4
0.5 mg Chit@MnFe_2_O_4_	600	0.36	-
0.5 mg MnFe_2_O_4_	720	0.26	-
Without catalyst	1 day	0.000018	-

* (a) 4-NP and (b) 4-NA stands for 4-nitrophenol and 4-nitroaniline, respectively.

## Data Availability

Not applicable.
